# Supplementary material for: A Comprehensive Evaluation of Metabolomics Data Preprocessing Methods for Deep Learning
Source: Metabolites. 2022 Feb 24;12(3):202. doi: 10.3390/metabo12030202 (PMC8948616; doi:10.3390/metabo12030202)
Supplement: Supplementary file 1 [file metabolites-12-00202-s001.zip › metabolites-1552727-supplementary caption .pdf]

metabolites

MDPI

Supplementary Materials:

Table S1: Classification results for Dataset 1 using a feedforward MLP with a hidden layer size of 8 units as described in the Methods section.

- The “label” column provides an abbreviated description of the model.
- The “Training_Train_Error_itersToValue” column provides the number of training iterations required to reach the minimum model error on the training dataset.
- The “Validation_Test_Error_itersToValue” column provides the number of training iterations required to reach the minimum model error on the test dataset.
- The “Training_AccuracyMCMicro_itersToValue” column provides the number of training iterations required to reach the maximum model accuracy on the training dataset.
- The “Validation_AccuracyMCMicro_itersToValue” column provides the number of training iterations required to reach the maximum model accuracy on the test dataset.
- The “Training_Train_Error” column provides the model error on the training data.
- The “Validation_Test_Error” column provides the model error on the test data.
- The “Training_AccuracyMCMicro” column provides the model accuracy on the training data.
- The “Validation_AccuracyMCMicro” column provides the model accuracy on the test data.
- The “Training_PrecisionMCMicro” column provides the model precision on the training data.
- The “Validation_PrecisionMCMicro” column provides the model precision on the test data.
- The “BN” column provides the abbreviation for the biomass normalization method used.
- The “Impute” column provides the abbreviation for the missing value imputation method used.
- The “Trans” column provides the abbreviation for the data transformation method used.
- The “Rep” column provides the model replicate number.

Table S2: Classification results for Dataset 2 using a feedforward MLP with a hidden layer size of 8 units as described in the Methods section. The table columns are the same as in Table S1.

Table S3: Classification results for Dataset 2 using a feedforward MLP with a hidden layer size of 2 units. All other parameters are the same as those described in the Methods section. The table columns are the same as in Table S1.

Table S4: Reconstruction results for Dataset 1 using a VAE architecture as described in the Methods section. The table columns correspond to the following:

- The “label” column provides an abbreviated description of the model.
- The “Training_Train_Error_itersToValue” column provides the number of training iterations to reach the minimum model error on the training data.
- The “Validation_Test_Error_itersToValue” column provides the number of training iterations to reach the minimum model error on the test data.
- The “Training_Train_Error” column provides the model error on the training data.
- The “Validation_Test_Error” column provides the model error on the test data.
- The “Training_CosineSimilarity-Mean” column provides the model mean cosine similarity score on the training data.
- The “Validation_CosineSimilarity-Mean” column provides the model mean using cosine similarity score on the test data.
- The “Training_CosineSimilarity-Var” column provides the model cosine similarity variance on the training data.
- The “Validation_CosineSimilarity-Var” column model cosine similarity variance on the test data.
- The “Training_PearsonR-Mean” column provides the model mean Pearson R score on the training data.
- The “Validation_PearsonR-Mean” column provides the model mean Pearson R score on the test data.
- The “Training_PearsonR-Var” column provides the model Pearson R variance on the training data.
- The “Validation_PearsonR-Var” column provides the model Pearson R variance on the test data.
- The “Training_EuclideanDist-Mean” column provides the model mean Euclidean Distance score on the training data.

- The "Validation_EuclideanDist-Mean" column provides the model mean Euclidean Distance score on the test data.
- The "Training_EuclideanDist-Var" column provides the model Euclidean Distance variance on the training data.
- The "Validation_EuclideanDist-Var" column provides the model Euclidean Distance variance on the test data.
- The "Training_ManhattanDist-Mean" column provides the model mean Manhattan Distance score on the training data.
- The "Validation_ManhattanDist-Mean" column provides the model mean Manhattan Distance score on the test data.
- The "Training_ManhattanDist-Var" column provides the model Manhattan Distance variance on the training data.
- The "Validation_ManhattanDist-Var" column provides the model Manhattan Distance variance on the test data.
- The "Training_JeffreysAndMatusitaDist-Mean" column provides the model mean Jeffrey's and Matusita Distance score on the training data.
- The "Validation_JeffreysAndMatusitaDist-Mean" column provides the model mean cosine similarity score on the training data.
- The "Training_JeffreysAndMatusitaDist-Var" column provides the model Jeffrey's and Matusita Distance variance on the training data.
- The "Validation_JeffreysAndMatusitaDist-Var" column provides the model Jeffrey's and Matusita Distance variance on the test data.
- The "Training_LogarithmicDist-Mean" column provides the model mean Logarithmic Distance score on the training data.
- The "Validation_LogarithmicDist-Mean" column provides the model mean Logarithmic Distance score on the test data.
- The "Training_LogarithmicDist-Var" column provides the model Logarithmic Distance variance on the training data.
- The "Validation_LogarithmicDist-Var" column provides the model Logarithmic Distance variance on the test data.
- The "Training_PercentDifference-Mean" column provides the model mean Absolute Percent Difference score on the training data.
- The "Validation_PercentDifference-Mean" column provides the model mean Absolute Percent Difference score on the test data.
- The "Training_PercentDifference-Var" column provides the model Absolute Percent Difference variance on the training data.
- The "Validation_PercentDifference-Var column provides the model Absolute Percent Difference variance on the test data.

Table S5: Reconstruction results for Dataset 2 using a VAE architecture as described in the Methods section. The table columns are the same as in Table S4.

Table S6: Supervised latent space disentanglement of class and style attribute reconstruction and classification results for Dataset 1 using the JointVAE architecture as described in the Methods section. The table columns correspond to the following:

- The "label" column provides an abbreviated description of the model.
- The "Training_Train_Error_itersToValue" column provides the number of training iterations required to reach the minimum model error on the training dataset.
- The "Validation_Test_Error_itersToValue" column provides the number of training iterations required to reach the minimum model error on the test dataset.
- The "Training_Train_Error" column provides the model error on the training data.
- The "Validation_Test_Error" column provides the model error on the test data.
- The "Training_CosineSimilarity-Mean" column provides the model mean cosine similarity score on the training data.
- The "Validation_CosineSimilarity-Mean" column provides the model mean using cosine similarity score on the test data.
- The "Training_CosineSimilarity-Var" column provides the model cosine similarity variance on the training data.
- The "Validation_CosineSimilarity-Var" column model cosine similarity variance on the test data.

- The "Training_PearsonR-Mean" column provides the model mean Pearson R score on the training data.
- The "Validation_PearsonR-Mean" column provides the model mean Pearson R score on the test data.
- The "Training_PearsonR-Var" column provides the model Pearson R variance on the training data.
- The "Validation_PearsonR-Var" column provides the model Pearson R variance on the test data.
- The "Training_EuclideanDist-Mean" column provides the model mean Euclidean Distance score on the training data.
- The "Validation_EuclideanDist-Mean" column provides the model mean Euclidean Distance score on the test data.
- The "Training_EuclideanDist-Var" column provides the model Euclidean Distance variance on the training data.
- The "Validation_EuclideanDist-Var" column provides the model Euclidean Distance variance on the test data.
- The "Training_ManhattanDist-Mean" column provides the model mean Manhattan Distance score on the training data.
- The "Validation_ManhattanDist-Mean" column provides the model mean Manhattan Distance score on the test data.
- The "Training_ManhattanDist-Var" column provides the model Manhattan Distance variance on the training data.
- The "Validation_ManhattanDist-Var" column provides the model Manhattan Distance variance on the test data.
- The "Training_JeffreysAndMatusitaDist-Mean" column provides the model mean Jeffrey's and Matusita Distance score on the training data.
- The "Validation_JeffreysAndMatusitaDist-Mean" column provides the model mean cosine similarity score on the training data.
- The "Training_JeffreysAndMatusitaDist-Var" column provides the model Jeffrey's and Matusita Distance variance on the training data.
- The "Validation_JeffreysAndMatusitaDist-Var" column provides the model Jeffrey's and Matusita Distance variance on the test data.
- The "Training_LogarithmicDist-Mean" column provides the model mean Logarithmic Distance score on the training data.
- The "Validation_LogarithmicDist-Mean" column provides the model mean Logarithmic Distance score on the test data.
- The "Training_LogarithmicDist-Var" column provides the model Logarithmic Distance variance on the training data.
- The "Validation_LogarithmicDist-Var" column provides the model Logarithmic Distance variance on the test data.
- The "Training_PercentDifference-Mean" column provides the model mean Absolute Percent Difference score on the training data.
- The "Validation_PercentDifference-Mean" column provides the model mean Absolute Percent Difference score on the test data.
- The "Training_PercentDifference-Var" column provides the model Absolute Percent Difference variance on the training data.
- The "Validation_PercentDifference-Var column provides the model Absolute Percent Difference variance on the test data.
- The "Training_AccuracyMCMicro" column provides the model accuracy on the training data.
- The "Validation_AccuracyMCMicro" column provides the model accuracy on the test data.
- The "Training_PrecisionMCMicro" column provides the model precision on the training data.
- The "Validation_PrecisionMCMicro" column provides the model precision on the test data.

Table S7: Supervised latent space disentanglement of class and style attribute reconstruction and classification results for Dataset 2 using the JointVAE architecture as described in the Methods section. The table columns are the same as in Table S6.

Table S8: Semi-supervised latent space disentanglement of class and style attribute reconstruction and classification results for Datasets 1 and 2 using the JointVAE architecture as described in the Methods section. The table columns are the same as in Table S6.

Table S9: Unsupervised latent space disentanglement of class and style attribute reconstruction results for Datasets 1 and 2 using the JointVAE architecture as described in the Methods section. The table columns correspond to the following:

- The "label" column provides an abbreviated description of the model.
- The "Training_Train_Error_itersToValue" column provides the number of training iterations to reach the minimum model error on the training data.
- The "Validation_Test_Error_itersToValue" column provides the number of training iterations to reach the minimum model error on the test data.
- The "Training_Train_Error" column provides the model error on the training data.
- The "Validation_Test_Error" column provides the model error on the test data.
- The "Training_CosineSimilarity-Mean" column provides the model mean cosine similarity score on the training data.
- The "Training_EuclideanDist-Mean" column provides the model mean Euclidean Distance score on the training data.
- The "Validation_EuclideanDist-Mean" column provides the model mean Euclidean Distance score on the test data.
- The "Training_EuclideanDist-Var" column provides the model Euclidean Distance variance on the training data.
- The "Validation_EuclideanDist-Var" column provides the model Euclidean Distance variance on the test data.
- The "Training_PercentDifference-Mean" column provides the model mean Absolute Percent Difference score on the training data.
- The "Validation_PercentDifference-Mean" column provides the model mean Absolute Percent Difference score on the test data.
- The "Training_PercentDifference-Var" column provides the model Absolute Percent Difference variance on the training data.
- The "Validation_PercentDifference-Var column provides the model Absolute Percent Difference variance on the test data.

Table S10: Latent space classification results for Dataset 1 using the JointVAE architecture as described in the Methods section. The table columns correspond to the following:

- The "label" column provides an abbreviated description of the model.
- The "category" column provides the TRUE classification category.
- The "predicted" column provides the predicted classification category.
- The "input" provides the abbreviation for the sample targeted for prediction.
- The "Training_Precision" column provides the precision of the classification prediction.

Table S11: Latent space classification results for Dataset 2 using the JointVAE architecture as described in the Methods section. The table columns are the same as in Table S10.

Table S12: Latent space traversal results for Dataset 1 using the JointVAE architecture and evaluation procedure as described in the Methods section. The table columns correspond to the following:

- The "label" column provides an abbreviated description of the model.
- The "step" column provides the discretized step in the continuous dimensions.
- The "gaussian_node" column provides the continuous node which is traversed according to the "step" column.
- The "categorical_node" column provides the discrete node selected.
- The "input" column is the expected class label.
- The "metric_value" column provides the value of the evaluation metric used to compute reconstruction similarity.
- The "metric" column provides the metric used to compute the reconstruction similarity.

Table S13: Latent space traversal results for Dataset 2 using the JointVAE architecture and evaluation procedure as described in the Methods section. The table columns are the same as in Table S12.
